# Deciphering Novel Transcriptional Regulators of Soybean Hypocotyl Elongation Based on Gene Co-expression Network Analysis

**DOI:** 10.3389/fpls.2022.837130

**Published:** 2022-02-22

**Authors:** Zhikang Shen, Min Chen

**Affiliations:** ^1^College of Life Science and Technology, Huazhong Agricultural University, Wuhan, China; ^2^State Key Laboratory of Crop Stress Adaptation and Improvement, School of Life Sciences, Henan University, Kaifeng, China; ^3^Academy for Advanced Interdisciplinary Studies, Henan University, Kaifeng, China

**Keywords:** gene co-expression network, hypocotyl elongation, auxin, light, transcription factor, soybean

## Abstract

Hypocotyl elongation is the key step of soybean seed germination, as well an important symbol of seedling vitality, but the regulatory mechanisms remain largely elusive. To address the problem, bioinformatics approaches along with the weighted gene co-expression network analysis (WGCNA) were carried out to elucidate the regulatory networks and identify key regulators underlying soybean hypocotyl elongation at transcriptional level. Combining results from WGCNA, yeast one hybridization, and phenotypic analysis of transgenic plants, a cyan module significantly associated with hypocotyl elongation was discerned, from which two novel regulatory submodules were identified as key candidates underpinning soybean hypocotyl elongation by modulating auxin and light responsive signaling pathways. Taken together, our results constructed the regulatory network and identified novel transcriptional regulators of soybean hypocotyl elongation based on WGCNA, which provide new insights into the global regulatory basis of soybean hypocotyl elongation and offer potential targets for soybean improvement to acquire cultivars with well-tuned hypocotyl elongation and seed germination vigor.

## Introduction

The consistency of seed germination and emergence is vital for large-scale agriculture, which not only reduces production cost but also increase yield potential. For dicots like soybean, hypocotyl elongation, the later stage of seed germination, determines whether the seedlings can break the soil smoothly and simultaneously, thus affecting the field emergence rate and consistency. Lots of research effort has focused on deciphering the regulation mechanism of hypocotyl development, mainly in the model species *Arabidopsis thaliana*. Similar to soybean, hypocotyl elongation in *Arabidopsis thaliana* is mainly driven by cell elongation. Effects of external environmental factors (light, temperature, gravity, etc.) as well endogenous hormones on hypocotyl elongation were well-studied (Deepika and Singh, 2020; Jiang et al., 2020). Generally speaking, the current research results show that the internal and external signals are integrated through PHYTOCHROME INTERACTING FACTORs (PIFs) to regulate hypocotyl elongation (Leivar et al., 2008; Shen et al., 2008; Castillon et al., 2009; Liu et al., 2011; Wang and Wang, 2015; Kim et al., 2016).

Before the seeds germinate and break the soil, they experience the process of hypocotyl elongation, curved top and leaf yellowing (chloroplast undeveloped), which is called “skotomorphogenesis.” Among them, hypocotyl elongation plays a key role in seedling emergence (Alabadi et al., 2004; Josse and Halliday, 2008; Yi et al., 2020). Once exposed to light, hypocotyl elongation is inhibited. So far, a variety of photoreceptors have been found to be involved in the regulation of hypocotyl elongation (Yu et al., 2013; Srivastava et al., 2015; Hu et al., 2021; Lin et al., 2021; Zhang et al., 2021; Zhong et al., 2021). For example, mutations of phytochrome A (PHYA) and phytochrome B (PHYB) caused insensitivity to far-red and red light in terms of the inhibition of hypocotyl elongation response (Seo et al., 2009; Lee et al., 2012; Zhong et al., 2012), indicating that PHYA and PHYB mediate the inhibition of hypocotyl elongation by far-red light and red light, respectively. Similarly, cryptochrome *cry1* mutant showed resistance to blue light induced inhibition of hypocotyl elongation, indicating that CRY1 mediated the inhibitory effect of blue light on hypocotyl elongation.

PIFs are basic helix-loop-helix (bHLH) transcription factors (TFs) that can interact with phytochrome proteins. They contain APA motifs which are capable of binding to PHYA or APB motifs binding to PHYB. So far, it has been reported that at least seven PIFs (1, 3, 4, 5, 6, 7, and 8) can interact with PHYA or PHYB (Leivar et al., 2009, 2012; Shin et al., 2009; Nozue et al., 2011; Hornitschek et al., 2012; Li et al., 2012; Oh et al., 2012; Jeong and Choi, 2013; Zhang et al., 2013). PHYA and PHYB can induce the phosphorylation and rapid degradation of their target PIFs through the interaction, while PIFs as TFs usually bind to the G-Box motif (CACGTG) in the promoter region of their downstream targets, such as *HISTONE DEACETYLASE 15* (*HDA15*) and *TIMING OF CAB EXPRESSION1* (*TOC1*), and regulate their expression to promote hypocotyl elongation. Therefore, light signal promotes the degradation of PIFs by activating the activity of PHYA/PHYB, so as to inhibit hypocotyl elongation. Interestingly, PIFs are also involved in multiple hormone signaling pathways to control hypocotyl elongation. For example, PIF3 is activated by ETHYLENE INSENSITVE3 (EIN3) and EIN3-LIKE1 (EIL1) in ethylene signaling pathway, as well act downstream of gibberellin (GA) signaling pathway, to promote hypocotyl elongation. Therefore, PIFS are the key regulators of hypocotyl elongation (Hyun and Lee, 2006; Mara et al., 2010; Castelain et al., 2012; Sassi et al., 2013; Gommers et al., 2017).

Almost all plant hormones are directly or indirectly involved in the regulation of hypocotyl elongation (Reed et al., 2018). It has been found that in *Arabidopsis thaliana*, auxin, GA or brassinosteroids (BRs) can promote the hypocotyl elongation, while abscisic acid (ABA) and cytokinin (CK) inhibit the process. For auxin, the expression of cell wall-related *EXPANSIN* genes was induced in hypocotyl grown under dark condition by auxin treatment (Pelletier et al., 2010; Cosgrove, 2016; Majda and Robert, 2018). A recent study showed that auxin induces the interaction between transmembrane kinase (TMK) and H^+^-ATPases in the plasma membrane, which facilitate proton transport from intracellular to cell wall, where EXPANSINs are activated to loosen and expand cell wall structure to allow the cell elongating in hypocotyl (Hocq et al., 2017; Ivakov et al., 2017; Duman et al., 2020; Xin et al., 2020; Lin et al., 2021). The role of ethylene (ET) is slightly complicated: it inhibits hypocotyl elongation under dark conditions and promotes elongation under light conditions (Jin et al., 2021). Studies have shown that ethylene acts through EIN3 to activate two opposite pathways (Paque et al., 2014), one mediated by PIF3 (phytochrome interacting factor 3) to promote hypocotyl elongation under light, and the other through ERF1 (ETHYLENE RESPONSE FACTOR1) to inhibit hypocotyl elongation under dark.

Soybean (*Glycine max*) is one of the most important economic crops in the world because of its high oil and protein concentrations (Liu et al., 2020). Similar to *Arabidopsis*, the elongation of soybean hypocotyl is affected by environmental factors (temperature, light, soil hardness, etc.) and phytohormones (auxin, GA, BRs, etc.) (Wang et al., 2021), but the underlying molecular mechanisms remain largely unclear. Studies have shown that soybean and *Arabidopsis thaliana* may have similar mechanisms of hypocotyl elongation. For example, a recent study showed that low light and high temperature promote soybean hypocotyl elongation through elevating the biosynthesis of auxin and GA, which is similar to that in *Arabidopsis thaliana* (de Lucas et al., 2008; Bawa et al., 2020). However, soybean has a genome size of 1.1–1.15 GB, which is much larger than that of *Arabidopsis thaliana*, implying soybean has more complicated regulatory networks underlying hypocotyl elongation. In order to elucidate the global architecture of the regulatory networks of soybean hypocotyl elongation, this study adopted the weighted gene co-expression network analysis (WGCNA) integrated with bioinformatics approaches. WGCNA was designed to find clusters (modules) of highly related genes, and correlate modules with external sample traits, so as to identify candidate regulators. Our study integrated the results of various big data analysis and obtained two transcriptional regulatory submodules and multiple novel candidate genes for hypocotyl elongation regulation. Our findings provide suitable combination of genes for the use of molecular breeding to produce soybean varieties with high germination rate and yield potential in the field.

## Materials and Methods

### Plant Materials and Growth Conditions

For soybean, cultivar Williams 82 was used as research material unless specified. For *Arabidopsis thaliana*, Columbia-0 ecotype was used as WT. The *Arabidopsis* T-DNA insertion line were obtained from Arashare (https://www.arashare.cn). All *Arabidopsis* T-DNA insertion mutants used in this study were listed in Supplementary Table 3. The T-DNA insertion liens were verified according to the method provided by the Salk Institute (http://signal.salk.edu/tdnaprimers.2.html). The primers used for T-DNA insertion verification were listed in Supplementary Table 6. All seeds were collected and stored under the same conditions.

For hypocotyl length analysis of *Arabidopsis* mutant lines, *Arabidopsis* seeds were surface-sterilized with 70% ethanol for 10 min, followed by four times washing with sterile-deionized water, and then grown on half-strength Murashige and Skoog (MS) medium (0.9% agar, pH 5.7), with or without a 16 h light/8 h dark photoperiod (light density 100 μmol m^−2^ s^−1^) at 22°C in a plant growth chamber. Hypocotyl length was determined in 5-day-old seedlings.

For phenotypic analysis of soybean seedlings, soybean seeds were surface-sterilized with 5% Sodium hypochlorite for 5 min, followed by four times washing with sterile-deionized water, and then germinated on filter papers for 2 d. The well-germinated seeds were planted in the vermiculite medium, and grown in a plant growth chamber with or without continuous white light (light density at 200 μmol m^−2^ s^−1^) at 27°C. Hypocotyl length was determined in 5-day-old seedlings.

### Exogenous Application of Phytohormone

The uniform seedlings (3-day-old, grown under continuous white light) were selected for phytohormone treatment. Seedlings were treated with phytohormone as follows: 10 μM IAA (Sangon, Shanghai, China), or 100 μM Kyn (Merck, Darmstadt, Germany), or water as control. The hypocotyl samples were collected after 2 d treatment for hypocotyl length measurement (with Image-Pro Plus software) and 30 min/2 h for RNA extraction. All treatments were performed with at least three biological replicates.

### RNA Extraction and RT-qPCR Analyses

Total RNA was extracted from at least 200 mg soybean hypocotyl using a plant RNAprep Kit (TianGen, Beijing, China). RNA concentration and purity were checked using the Qubit RNA Assay Kit using Qubit 2.0 Fluorometer (Life Technologies, CA, USA). First-strand complementary DNA (cDNA) was synthesized with 2 μg total RNA using a Reverse Transcription kit (Vazyme, Nanjing, China) and 80 ng of first strand cDNA were used as the template for qPCR. The qPCR analyses were performed on a CFX96 Touch Real-Time PCR Detected System (BIO-RAD, CA, USA). The RNA levels were calculated as described before (Chen and Penfield, 2018). The reference gene was a soybean ACTIN gene (*GLYMA_02G091900*). Three biological replicates with three technical replicates were performed for each sample. Primer sequences are listed in Supplementary Table 6.

### RNA Sequencing

Total RNA was extracted from at least 250 mg soybean hypocotyl and root tip samples, respectively. RNA integrity was assessed using the RNA Nano 6000 Assay Kit of the Bioanalyzer 2100 system (Agilent Technologies, CA, USA). Sequencing libraries were generated with 400 ng total mRNA using the NEB RNA Library Prep Kit for Illumina (NEB, MA, USA), and for quality control using Agilent 2100 bioanalyzer. All libraries were sequenced in paired-end 150 bases protocol (PE150) on an Illumina NovaSeq 6000 system (Illumina, CA, USA).

Quality control of sequencing data was carried out with fastp. The raw reads were then trimmed to discard adapter sequences, reads containing poly-N and with low quality by using TrimGalore. After that, 6.19–9.12 GB clean reads were obtained for further analysis. Clean reads were mapped to the soybean reference genome (Williams 82 V2.1) using Hisat2 with default parameters. Number the reads mapped to each gene was calculated with featureCounts. And then fragments per kilobase of transcript per million mapped reads (FPKM) of each gene was calculated based on the length of the gene and reads count mapped to this gene using R. All raw reads were deposited to the National Center for Biotechnology Information Sequence Reads Archive (SRA) with accession number: PRJNA789395, PRJNA789276, and PRJNA790743.

### Regulatory Network Construction by WGCNA

WGCNA was performed to identify gene co-expression networks associated with hypocotyl elongation by using the WGCNA software package as previously described (Langfelder and Horvath, 2008). Briefly, after excluding genes with missing entries or average FPKM <1, 18 samples and 23,729 genes were selected to construct the weighted gene co-expression network. The correlation matrix was then constructed using pairwise Pearson Correlations among all genes. The soft threshold was set to β = 24 to achieve a scale-free network. To identify gene modules, the dynamic tree-cutting algorithm was used. Gene modules with similar expression profiles were merged (PCC > 0.85). Gene connectivity in each module was determined, and hub genes were identified as the genes with the highest connectivity. The global gene co-expression network was visualized with Cytoscape_v3.5.1 (Shannon et al., 2003).

To identify modules correlated with hypocotyl elongation, a network-weighted gene significance function was used. Gene significance (GS) was defined as the correlation between a specific gene expression level and the hypocotyl development. Gene modules associated with hypocotyl elongation were also identified based on the correlation between Module eigengene (ME) and hypocotyl development. ME is considered representative expression of all genes in one module and was identified using the ME function.

To analyse functional annotation of the identified genes, all genes in the candidate modules were subjected to gene ontology (GO) functional annotation and Kyoto Encyclopedia of Genes and Genomes (KEGG) pathway enrichment analyses using the ClusterProfiler tool and KEGG website (https://www.genome.jp/kegg) (Yu et al., 2012). Functional enrichment was performed in the following three GO categories: biological process (BP), molecular function (MF), and cellular component (CC).

### Cis-Motif Enrichment Analysis

Motif discovery was performed with the MEME suite (https://meme-suite.org/), using the 2.5 kb sequence upstream of the putative target genes' ATG start codon. The TOMTOM tool was exerted to predict the functions of the enriched motifs by comparing them with known cis-motifs.

### Creation of Transgenic Plants

To generate *GmPRE6* (*Glyma.01g044800 and Glyma.08g298700*)-overexpression lines, the full-length coding sequence (CDS) of the two *GmPRE6s* was amplified and cloned into the the pCambia1300-eCFP vector separately. The constructs was then introduced into Agrobacterium strain GV3101 and transformed into *Arabidopsis thaliana* plants by floral infiltration. The T1 transgenic plants were selected on hygromycin-containing medium, and the progeny were used for subsequent experiments.

### Agroinfiltration of *N. sbenthamiana*

The pCambia1300-eCFP plasmid that harboring *GmPRE6* CDS (driven by the *CaMV 35S* promoter) were mobilized into GV3101. Recombinant agrobacteria were prepared for infiltration using a modified protocol published before. In short, recombinant bacteria cultures were incubated overnight at 28 °C with shaking. Bacteria were pelleted by centrifugation (14,000 g for 5 min) and resuspended to an OD600 = 1.0 in MMA (10 mM MES pH 5.6, 10 mM MgCl_2_, 200 μM acetosyringone). Bacteria were delivered into the underside of young leaves using a blunt tipped plastic syringe and applying gentle pressure. *N. benthamiana* leaf samples were collected 24–48 h post agroinfiltration and the fluorescence was imaging using a Leica confocal microscope (LSM 900, Zeiss, Germany).

### Yeast One-Hybrid Assay

Y1H assays were performed following a previously published method (Fuxman Bass et al., 2016) with minor modifications. Briefly, a 370-bp, a 200-bp, and a 643-bp promoter fragment of *Glyma.12G034600, Glyma.12G033900*, and *Glyma.11G031700* was amplified (Supplementary Table 6) and cloned into the pAbAi-bait vector, respectively. The pAbAi-bait plasmids were subsequently linearized and transformed into yeast (Y1H Gold strain). The transformed colonies were screened by colony PCR. The prey constructs (pGADT7-TFs) were transformed into yeast harboring a pAbAi-bait plasmid and screened on the SD-Leu/AbA plate. The minimum inhibitory concentration of aeurobasidin A was 200 ng/mL. At least three independent biological replicates were performed for each combination.

## Results

### A Hub Module Regulating Hypocotyl Elongation Identified Using WGCNA

**Figure 1:**
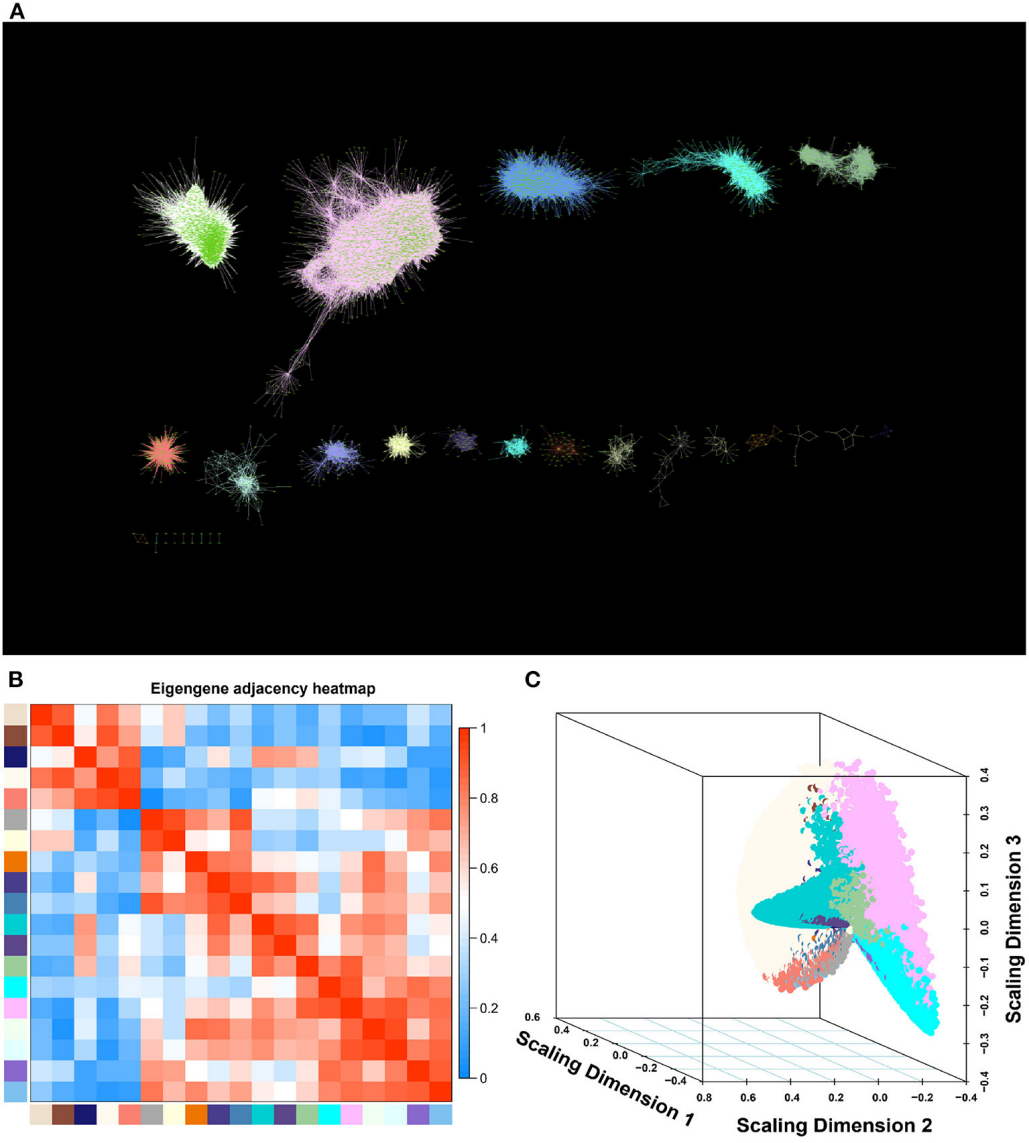
A global gene co-expression network related to hypocotyl length in soybean. **(A)** Global gene co-expression network including 19 modules visualized with Cytoscape. **(B)** Eigengene adjacency heatmap of 19 modules. Each row/column corresponds to a module. Each cell contains a corresponding correlation indicated by color. **(C)** Multidimensional scaling plots of the TOM-based dissimilarity of gene expression in different modules.

In order to explore the global regulatory mechanisms underlying soybean hypocotyl elongation, we profiled the transcriptomes of the emerging hypocotyl and root tip tissue at 0, 1, and 5 days after imbibition (DAI) in the soybean cultivar “Williams 82” using RNA-seq (Supplementary Figure 1). The root tip tissue was set as negative control since its elongation mode is largely different from that of hypocotyl (root tip has vigorous cell division but hypocotyl does not). Eighteen samples, 23,729 genes were screened and analyzed using WGCNA. After normalization, no outlier module was eliminated. The soft-thresholding power was set to β = 24 (scale free *R*^2^ = 0.92) to ensure a scale-free network (Supplementary Figure 2). We identified a total of 97 modules via average linkage hierarchical clustering (Figure 1A). The number of genes contained in these clusters ranged from 58 to 9,854 (Supplementary Table 1), and some of them were highly correlated between each other (Figure 1B). In order to simplify the analysis, we combined clusters with Pearson correlation coefficient (PCC) >0.85, and finally obtained 19 modules for subsequent analysis (Figure 1C; Supplementary Table 1).

**Figure 2:**
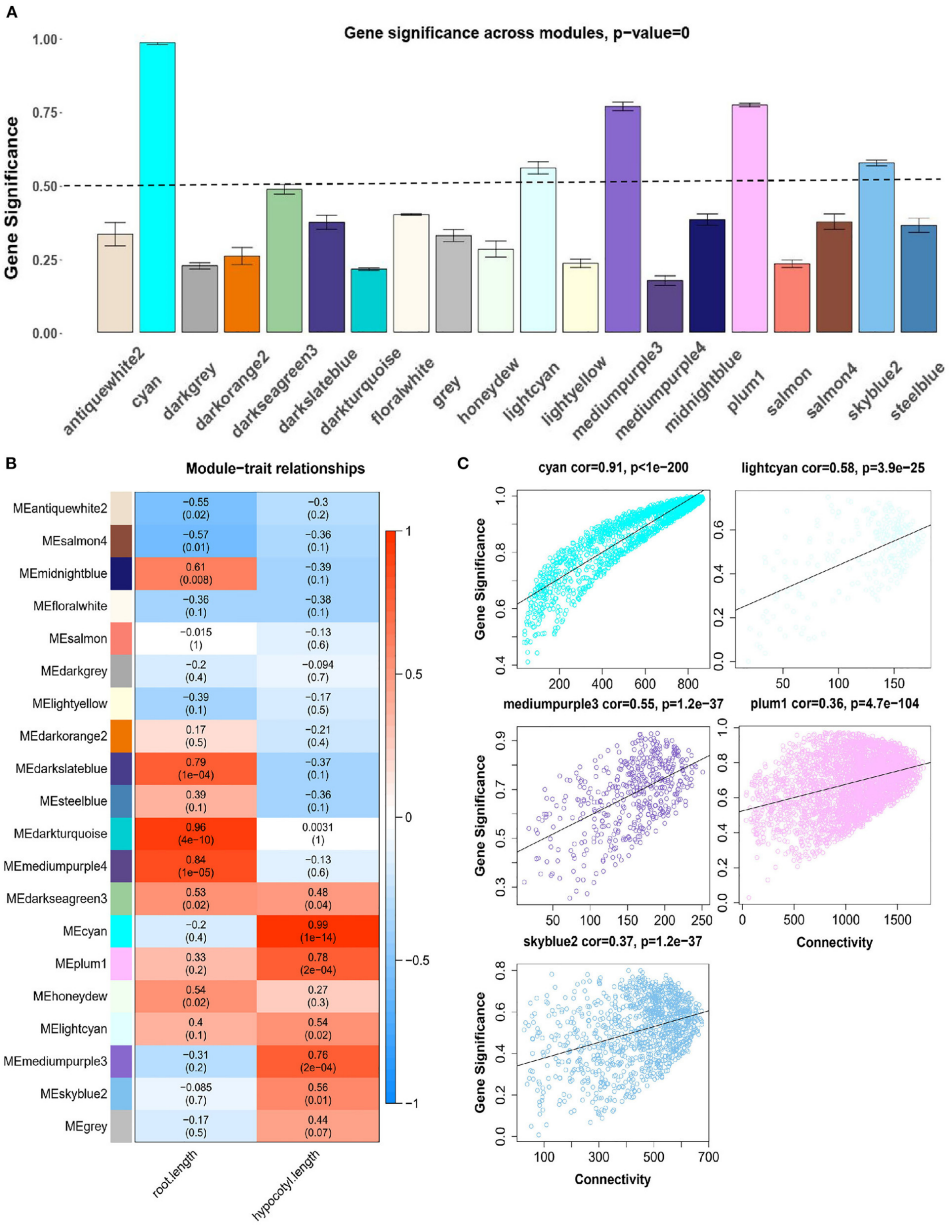
Correlation between modules and traits. **(A)** The mean gene significance between module and hypocotyl development. **(B)** Module-trait relationship. Each row corresponds to a module, while each column corresponds to the root/hypocotyl development trait. Each cell contains a corresponding correlation. **(C)** The correlation between gene significance and connectivity in the 5 modules with gene significance>0.5 in **(A)**.

Genes in the same module could form networks and may participate in similar biological processes, so we examined the relationship between modules and hypocotyl elongation phenotype. By calculating the correlation significance between gene expression level in every single module and hypocotyl elongation phenotype, we found five modules with average significance higher than 0.5, of which a cyan module reached 0.94 (Figure 2A; Supplementary Table 2), indicating it may participate in hypocotyl elongation. Further, we analyzed the correlation between module eigengene (ME, which reflects the overall gene expression level of the entity module) and hypocotyl elongation, and again found that cyan module was significantly correlated with hypocotyl elongation phenotype (*R* = 0.99; Figure 2B). Therefore, the cyan module is considered to be the hub module regulating hypocotyl elongation. Consistent with the above results, most of the genes in the cyan module were specifically highly expressed in the hypocotyl tissue at 5 DAI (Supplementary Figure 3), and the connectivity of the genes was significantly positively correlated with its significance (*R* = 0.91, Figure 2C).

To confirm that cyan module is indeed a regulation module of soybean hypocotyl elongation, we randomly selected six genes with high connectivity in the module (*Glyma.14g218800, Glyma.15g253700, Glyma.03g060300, Glyma.15g209000, Glyma.06g039900, Glyma.16g205200*, and obtained their *Arabidopsis* homolog knockout mutants (homolog inferred from NCBI blast program, and when there are multiple results, the one with the highest score was selected; *Arabidopsis* mutants purchased from AraShare https://www.arashare.cn). We found that four out of six *Arabidopsis* mutants showed altered hypocotyl elongation compared to wild type control (*hxk3*, corresponding to *Glyma.14g218800*; *lhcb5*, corresponding to *Glyma.16g205200*; *lhcb4.2*, corresponding to *Glyma.03g060300; emb1473*, corresponding to *Glyma.15g209000*) (Supplementary Figure 4; Supplementary Table 3), indicating that the cyan module is a convincible regulatory candidate of soybean hypocotyl elongation.

### Characterization of a Conserved *PIFs-SAURs-EXPANSINs* Submodule

**Figure 3:**
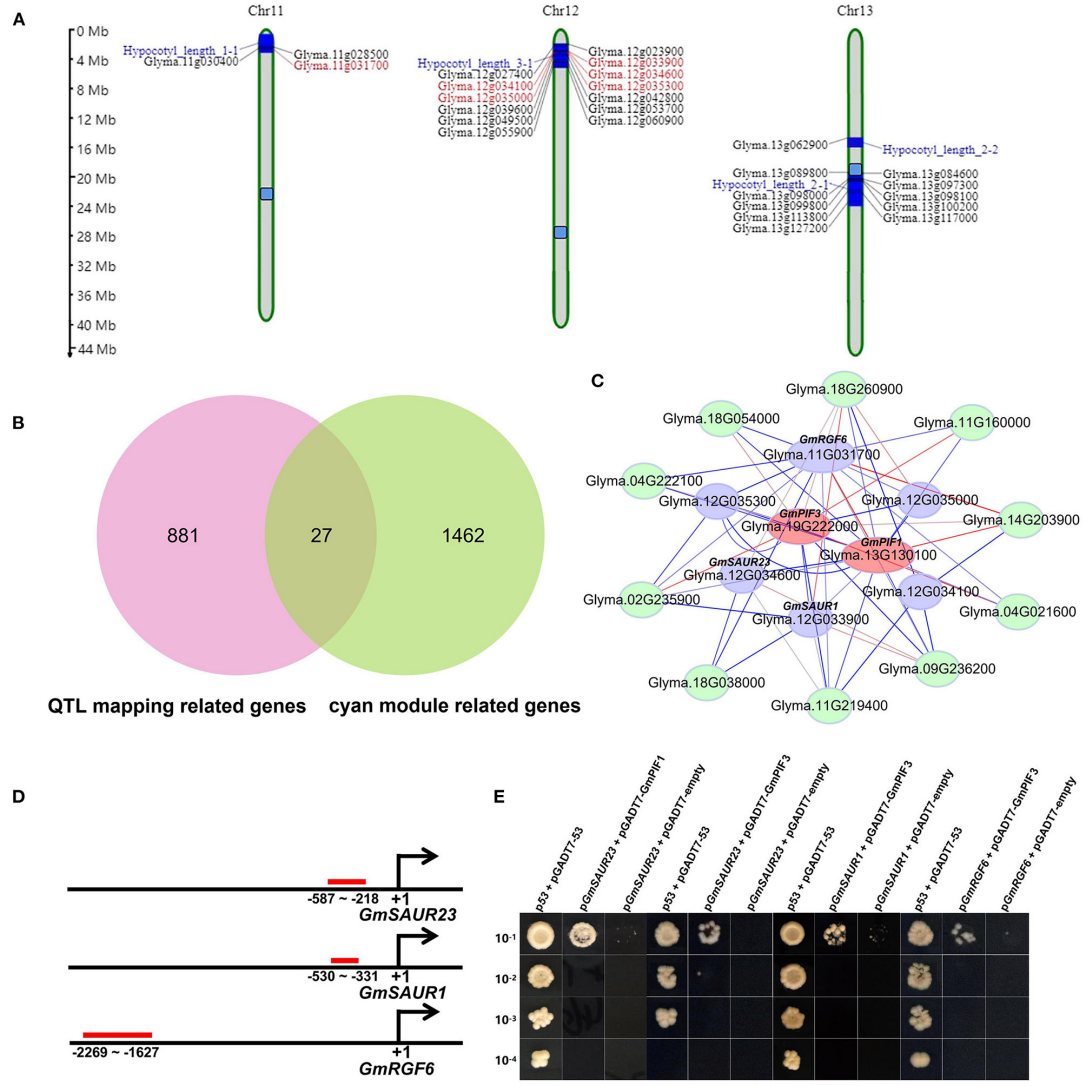
Construction of a *GmPIFs*-*GmSUAR*s/*GmRGF6* submodule regulating hypocotyl elongation. **(A)** Distribution of 27 common members between the cyan module and hypocotyl elongation related QTLs on chromosomes. The blue block indicates location of QTLs. The light bule block indicates position of centromeres. Genes in red are *GmSAUR*s and *GmRGF6*. **(B)** Venn diagram showing the intersection of the cyan module with hypocotyl elongation related QTLs. **(C)** Gene co-expression network in *GmPIFs*-*GmSUAR*s/*GmRGF6* submodule. Color of the lines indicates weight value: red indicates high value and blue indicates low value. **(D)** GmPIFs bind to the promoter regions of *GmSUARs* and **(E)** *GmRGF* revelaed by Y1H assays. Red lines indicated the potential binding regions of GmPIFs.

There are 1,532 genes in the cyan module. Gene Ontology biological process (GO-BP) analysis showed that the genes in the cyan module were mainly enriched in GO-BP terms related to auxin response, light response and plant cell wall structure (Supplementary Figure 6). Among them, the genes related to cell wall structure contain 10 *EXPANSIN*s, which might be involved in auxin induced cell wall expansion and hypocotyl elongation as previously reported (Zhao et al., 2008). These results were consistent with the previous reported mechanisms in *Arabidopsis thaliana*, which highlighted the regulation of hypocotyl elongation by auxin and light. Interestingly, when comparing the genes in cyan module with the four previously reported quantitative trait loci (QTLs) related to hypocotyl elongation (Lee et al., 2001; Liang et al., 2014), we found 27 genes appearing in both results (Figures 3A,B). To study whether and how these genes are involved in regulating hypocotyl elongation, the promoters of these genes and their homologous genes in *Arabidopsis thaliana* (homolog inferred from NCBI blast program; promoter spans 2-kb region upstream of the ATG translation start site) were scanned for cis-elements (MEME suite https://meme-suite.org/) (Bailey et al., 2009). Interestingly, the enriched elements included the one responsive to auxin (class I), the one responsive to light response (class II), etc. (Supplementary Figure 7), suggesting that these genes might be involved in the auxin and light regulating hypocotyl elongation process. It is worth noting that in these genes there are five small auxin up RNAs (SAURs) members. Previous studies have shown that SAURs act as PIFs' direct targets to participate in hypocotyl elongation in *Arabidopsis thaliana*. In the cyan module, there are two members of soybean PIF family, *GmPIF1* (*Glyma.13g130100*) and *GmPIF3* (*Glyma.19g222000*) (weighted value = 0.48). We found that *GmPIF1* and *GmPIF3* were of high connectivity with these five *GmSAURs* as well one other auxin responsive gene *GmRGF6* (*ROOT MERISTEM GROWTH FACTOR6, Glyma.11G031700*) in the 27 genes (weighted value = 0.51). Intriguingly, the 10 *EXPANSIN* genes related to cell wall growth in the cyan module were closely correlated with *GmSAURs* and *GmRGF6*. Based on these results, we constructed a novel submodule composed of *GmPIF1*/*3, GmSAURs, GmRGF6* and *EXPANSIN*s as the regulator of hypocotyl elongation (Figure 3C).

In *Arabidopsis thaliana* and other species, PIFs act through SAURs to regulate hypocotyl elongation in response to external and internal cues such as light, auxin and temperature. Through the analysis of previously published ChIP-seq data (Pfeiffer et al., 2014), we found that both AtPIF1 and AtPIF3 bind to the promoter regions of *AtSAUR1, AtSAUR23* and *AtRGF6* (Supplementary Table 4). GmPIF1 and GmPIF3 have high homology to AtPIF1 and AtPIF3 (Supplementary Figure 5), suggesting that GmPIF1 and GmPIF3 may act through the similar mechanism, i.e., binding to the promoters of *GmSAURs*. To test this hypothesis, the regions containing class I and II motifs (Supplementary Figure 7) in the promoter regions of *GmSAUR1, GmSAUR23*, and *GmRGF6* (Figure 3D) were selected as baits in yeast one hybridization analysis to identify interactors. Not surprisingly, it is found that GmPIF1 can bind to the promoter of *GmSAUR23*, and GmPIF3 can bind to the promoter of *GmSAUR23, GmSAUR1*, and *GmRGF6* (Figure 3E), indicating that the binding of PIFs to the promoter region of SAURs is indeed a conservative mechanism.

**Figure 4:**
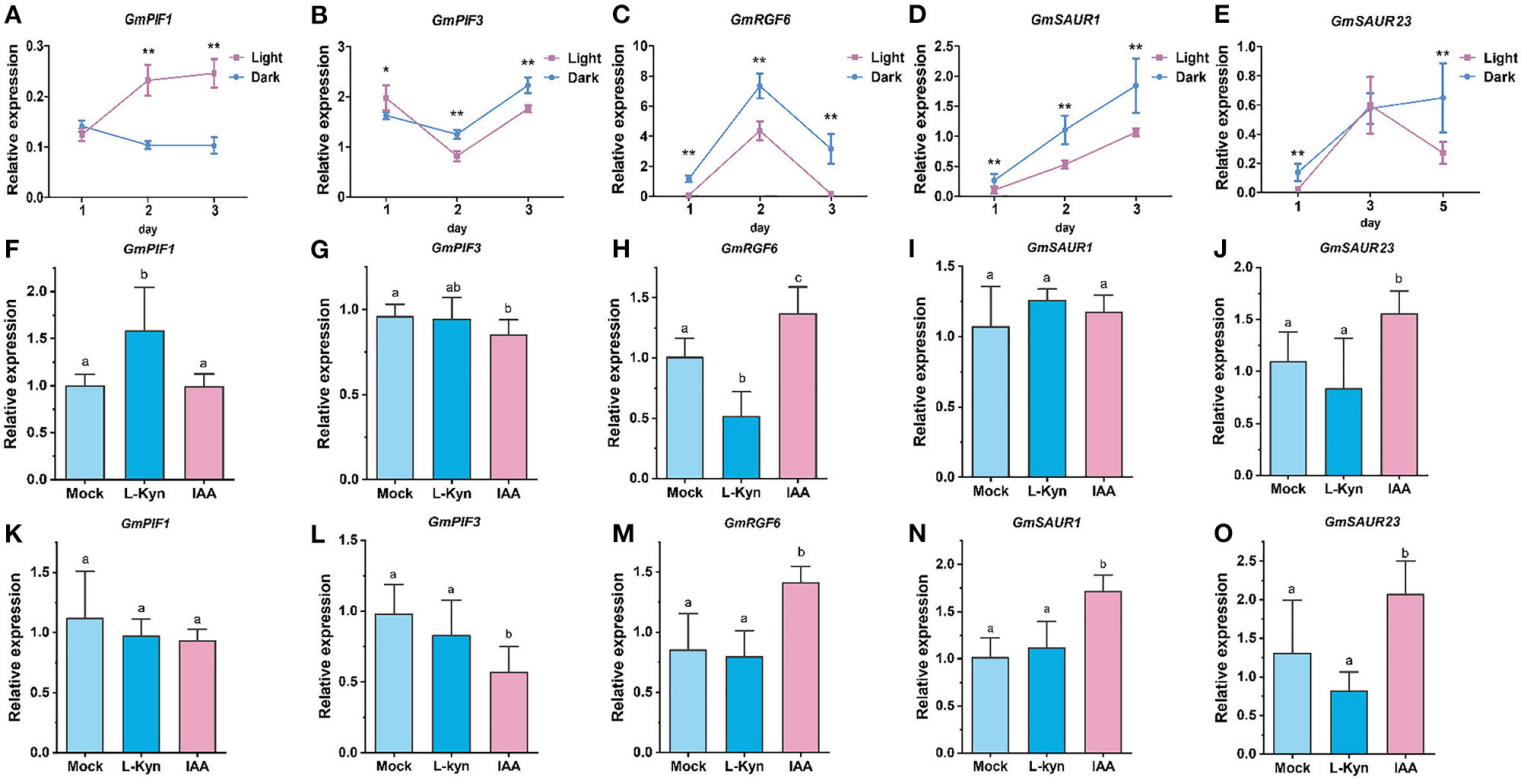
Experssion of *GmPIFs* and *GmSUAR*s/*GmRGF6* in response to light and exogenous auxin. **(A–E)** *GmPIFs* and *GmSUAR*s/*GmRGF6* expression in soybean hypocotyl under light and dark condition. **(F–J)** *GmPIFs* and *GmSUAR*s/*GmRGF6* expression in the hypocotyl after 30 min treatment of exogenous IAA and Kyn. **(K–O)** *GmPIFs* and *GmSUAR*s/*GmRGF6* expression in the hypocotyl after 2 h treatment of exogenous IAA and Kyn. Data are presented as mean ± SD (*n* = 3). A statistically significant difference of *p* < 0.05 or *p* < 0.01 relative to mock (two-tailed Student's *t*-test) is denoted by * and **, respectively. Different letters (a, b) indicate significant difference (two-tailed Student's *t-*test).

Auxin and light are important factors regulating hypocotyl elongation. Similar to the case for *Arabidopsis*, light inhibited the elongation of soybean hypocotyl (data not shown), while exogenous auxin promoted the elongation (Supplementary Figure 8). The above studies showed that PIFs-SAURs-EXPANSINs submodule may be involved in hypocotyl elongation regulated by light and auxin. To verify this, we examined the expression levels of *GmPIF1, GmPIF3, GmSAUR1, GmSAUR23*, and *GmRGF6* and two *EXPANSIN*s (*Glyma.14g203900* and *Glyma.02g235900*) under dark-light change and exogenous auxin by RT-qPCR (Figure 4; Supplementary Figure 9). The results show that the expression of *GmPIF1* was induced by light, while the expression of *GmPIF3* was first induced and then inhibited by light. Previous studies have shown that light mainly regulates AtPIF1 and AtPIF3 at the protein level (Qiu et al., 2017). Our results suggest that light may regulate the expression of PIFs at the transcriptional level in soybean, which reflects a feedback regulation mechanism. The expression of *GmSAUR1, GmSAUR23*, and *GmRGF6* was inhibited by light, while previous studies in *Arabidopsis thaliana* showed that light induced the expression of several other *SAUR*s (Dong et al., 2019), suggesting that SAUR family members may have functional differentiation among different species. When treated with exogenous indole-3-acetic acid (IAA) and L-Kynurenine (Kyn), an auxin biosynthesis inhibitor, the expression of *GmPIF1* and *GmPIF3* in the hypocotyl showed slight responses, which may be due to auxin mainly inhibiting the transcriptional activation activity of PIFs through downstream AUX/IAAs rather than regulating their expression directly. The expression of *GmRGF6* was induced by exogenous IAA and inhibited by Kyn, which is similar to the case in *Arabidopsis thaliana* (Busatto et al., 2017). The expression of *GmSAUR1* and *GmSAUR23* was similarly induced by exogenous IAA, but with no obvious response to Kyn. It should be noted that the expression of the two *EXPANSIN* genes was inhibited by light, and induced by exogenous IAA (Supplementary Figure 9). These results suggest that *GmSAUR1, GmSAUR23*, and *GmRGF6* may be positive regulators of soybean hypocotyl elongation. They perceive signals such as light and auxin and regulate hypocotyl elongation through downstream EXPANSINs.

### Characterization of a *GmPRE6*s Based Submodule

**Figure 5:**
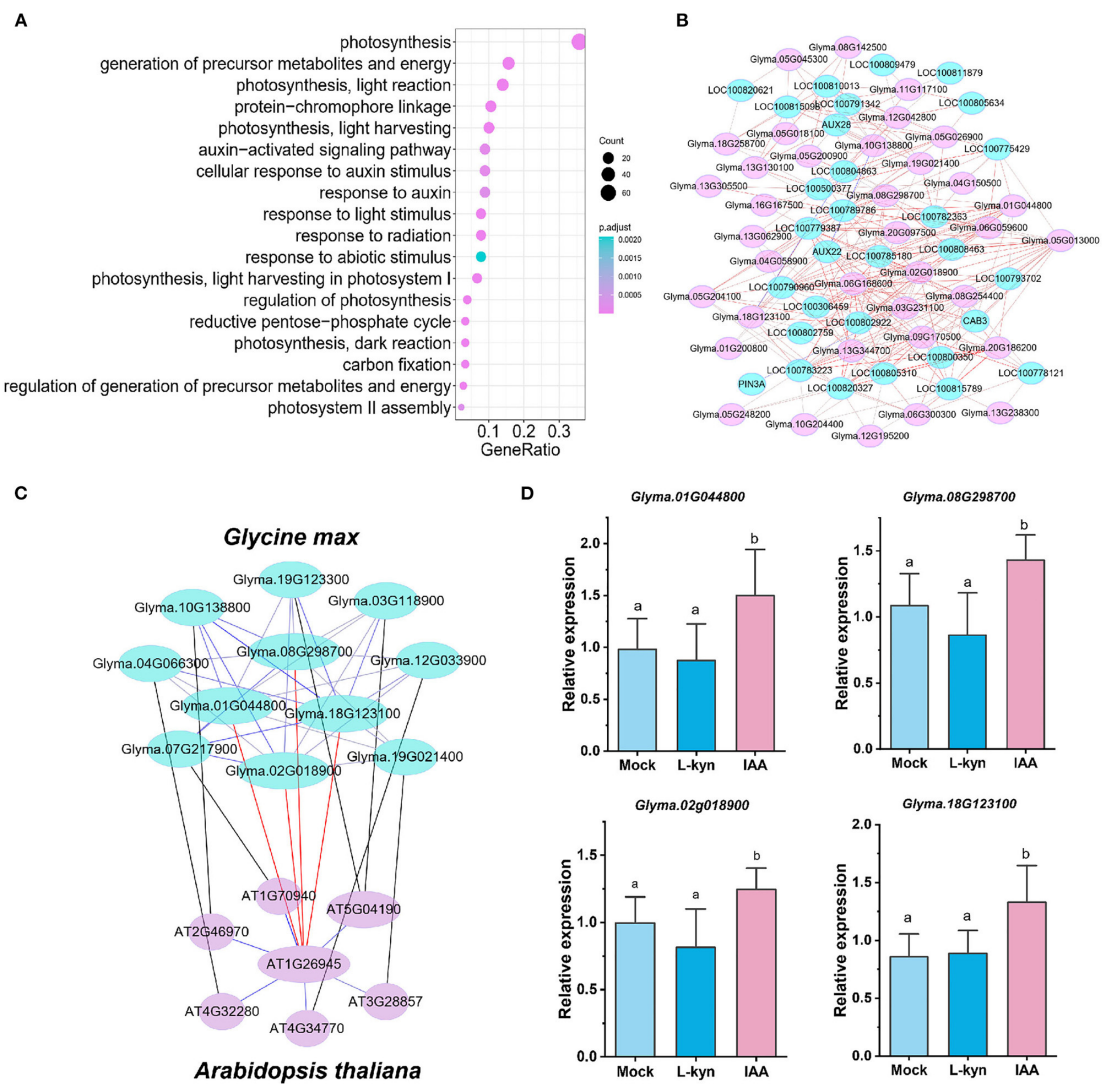
Construction of a *GmPRE6*s based submodule regulating hypocotyl elongation. **(A)** GO analysis of TFs in the cyan module. **(B)** Co-expression network containing light and auxin response TFs and their closely related genes in the cyan module. **(C)** A conserved PRE6 based submodule regulating hypocotyl elongation between *Glycine max* and *Arabidopsis thaliana*. **(D)** Expression of *GmPRE6s* in the hypocotyl after 2 h treatment of exogenous IAA and Kyn. Data are presented as mean ± SD (*n* = 3). Different letters (a, b) indicate significant difference (two-tailed Student's *t*-test).

Genes with similar expression pattern in a single module might be targets of a cluster of TFs involved in the same biological process (Zaidi et al., 2020). In order to identify the TFs which potentially regulate the hypocotyl elongation in the cyan module, we used PlantTFDB (planttfdb.gao-lab.org) to predict the TFs, and obtained 83 TFs of 24 families in the cyan module. Among them, the bHLH family transcription factors (Supplementary Figure 10) accounted for the largest proportion (22.9%). Gene Ontology biological process (GO-BP) analysis showed that many of these TFs were involved in the response to light and auxin (36/83, Figure 5A). We extracted these transcription factors and 85 closely connected genes to them (weighted value > 0.45) in the cyan module, and constructed a TF based submodule (Figure 5B). It is not surprising that the submodule includes multiple auxin responsive and light responsive genes (Supplementary Table 7), such as *PIN3A* (*AUXIN EFFLUX CARRIER COMPONENT3A, Glyma.07G217900*, involved in auxin polar transport), *AUX22* and *AUX28* (*AUXIN-INDUCED PROTEIN*22 and 28, *Glyma.08G207900*, and *Glyma.19G161100*, involved in auxin signal transduction) and *CAB3* (*CHLOROPHYLL A/B BINDING PROTEIN3, Glyma.05G128000*, responsive to light) etc., indicating this submodule act as well in a light and auxin response dependent manner.

Interestingly, in this submodule, there are four homologous genes of *Arabidopsis PACLOBUTRAZOL RESISTANCE6* (*PRE6*). PREs are atypical bHLH transcription factors that lack the basic DNA binding domain. Accumulated evidence suggests that PREs are involved in the regulation of hypocotyl elongation, including PRE6 (Hong et al., 2013; Zheng et al., 2017). We extracted genes of highest correlation with these four *GmPRE6*s (weighted value > 0.4) from cyan module and constructed a *GmPRE6*s based submodule. It is worth noting that the genes in this submodule are specifically highly expressed during hypocotyl development (Supplementary Figure 11), indicating their important roles in regulating hypocotyl elongation. Interestingly, in an *Arabidopsis thaliana* hypocotyl elongation related module obtained through WGCGA analysis, 27 samples, 33,557 genes, based on RNA-seq results from Burko et al. (2020) (Supplementary Figure 12), a homologous submodule was found, i.e., members between the two submodules are homologous genes. Among these genes in the two submodules, PRE6's potential downstream targets, *PIN3* (*At1g70940*-*Glyma.07g217900*), *PKS4* (*PHYTOCHROME KINASE SUBSTRATE4, At5g04190*-*Glyma.19g123300*/*Glyma.03g118900*), *IAA29* (*INDOLE-3-ACETIC ACID INDUCIBLE29, At4g32280*-*Glyma.04g066300*), *PRE5* (*At3g28857*-*Glyma.19g021400*), *SAUR1* (*At4g34770*-*Glyma.12g033900*) were all reported to be involved in auxin or light regulating hypocotyl elongation (Zheng et al., 2017; Majda and Robert, 2018; Dong et al., 2019; Hu et al., 2021). Interestingly, the 2.5 kb promoter regions of these genes all contain at least one G-box motif (potential binding site of PIFs) (Supplementary Figure 14E), indicating they are potential targets of PRE6 related PIFs. A previous study (Hong et al., 2013) showed that in *Arabidopsis* PRE6 forms non-functional heterodimers with LONG HYPOCOTYL IN FAR-RED1(HFR1) to activate PIF4 and induce hypocotyl elongation. We note that in the submodule Glyma.10g138800 (PHYTOCHROME INTERACTING FACTOR-LIKE15, GmPIL15) is the soybean homolog of *Arabidopsis* HFR1, so it is possible GmPRE6s interact with GmPIL15 to activate GmPIFs thus regulating hypocotyl elongation. These results suggest that PRE6 and its downstream genes constitute a conserved regulatory submodule involved in auxin and light regulating hypocotyl elongation (Figure 5C).

**Figure 6:**
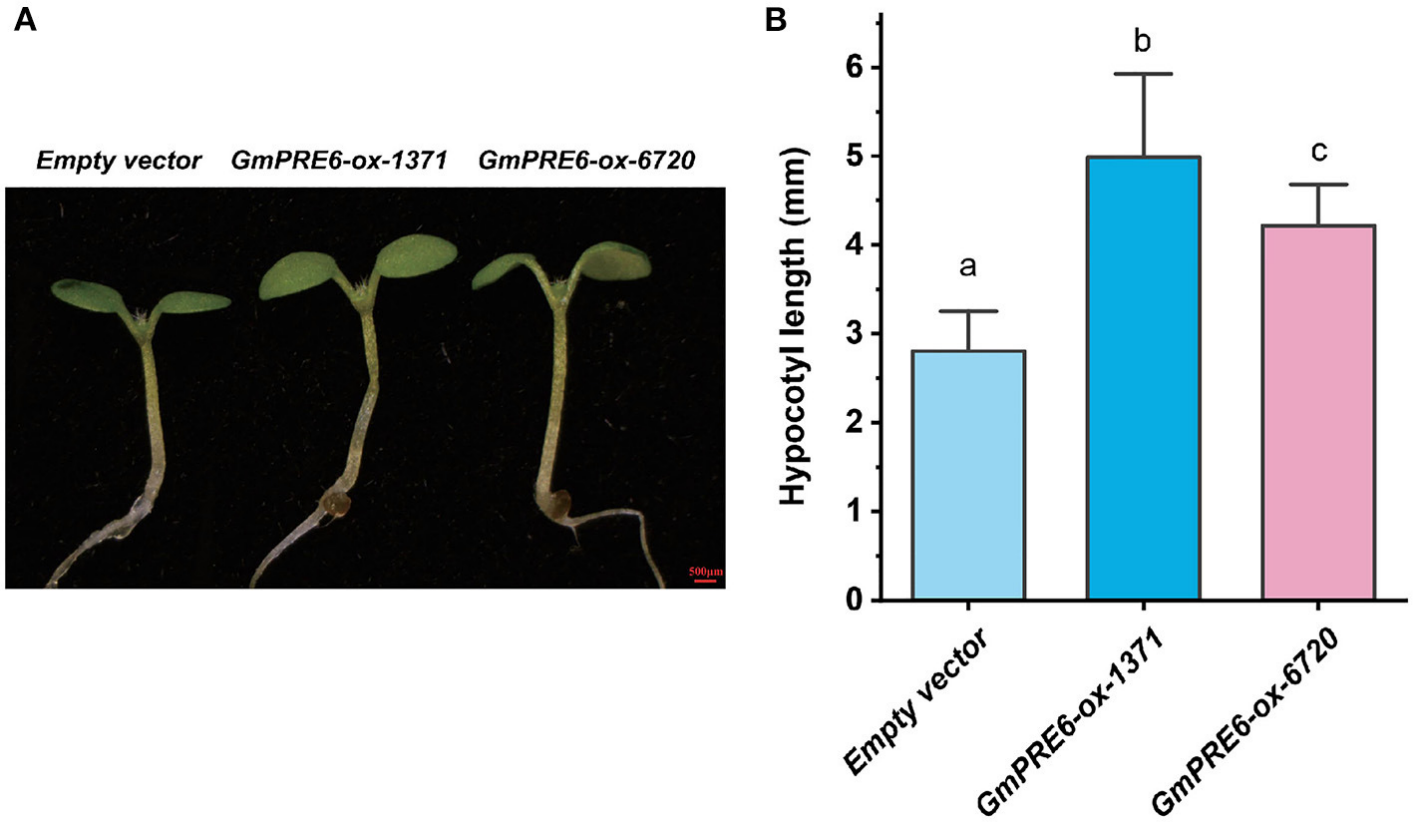
The hypocotyl phenotype of *Arabidopsis* seedlings overexpressing one *GmPRE6*. **(A)** Overexpression of the *GmPRE6* induces a long hypocotyl phenotype in transgenic seedlings. Seedlings were grown for 5 d under light condition. **(B)** Hypocotyl length measurement. Data are presented as mean ± SD (*n* = 13–19). Different letters (a, b) indicate significant difference (two-tailed Student's *t*-test).

The four GmPRE6s were expressed in the nucleus (Supplementary Figure 13). RT-qPCR results showed that their expression was induced by exogenous IAA (Figure 5D) and inhibited by light (Supplementary Figures 14A–D). Multiple light and auxin responsive elements can be found in the promoter region of *GmPRE6s* (Supplementary Figure 14E), which is consistent with the above results. In order to verify whether and how GmPRE6s was involved in the regulation of soybean hypocotyl elongation, we ectopically expressed two of *GmPRE6*s (*Glyma.01g044800* and *Glyma.08g298700*) in *Arabidopsis thaliana*, and the result showed that overexpression of *GmPRE6*s induced hypocotyl elongation (Figure 6). These results suggest that GmPRE6s may participate in hypocotyl elongation pathway regulated by auxin and light factor.

## Discussion

Constructing gene regulatory networks through WGCNA has been proved to be an effective approach to mine important phenotype related regulators. By conducting transcriptome analysis at 0, 1 and 5 DAI during soybean hypocotyl development and WGCNA analysis, we established the co-expression regulatory network of soybean hypocotyl elongation (Figure 1), and found a novel cyan module highly related to the phenotype (Figure 2). Most of the genes in this module are enriched in the pathways related to light response, auxin response, and cell wall expansion (Supplementary Figure 6). Thus, the cyan module might be involved in hypocotyl elongation mainly regulated by light and auxin.

Two aspects of evidence show that the cyan module is a reliable candidate of hypocotyl elongation regulation. On the one hand, four out of six mutants of cyan module members' homologous genes in *Arabidopsis thaliana* showed altered hypocotyl elongation phenotype (Supplementary Figure 4); on the other hand, there are 27 genes exist in both the cyan module and previously identified soybean hypocotyl elongation QTLs (Figures 3A,B). Interestingly, we found a conserved submodule that exists in both the cyan module and an *Arabidopsis* module related to hypocotyl elongation, indicating the conservation of hypocotyl elongation mechanism among species (Figure 5C).

In the 27 common genes between the cyan module and previously published QTLs, there are five *SAURs* (Figure 3C). SAURs were reported to be involved in the regulation of hypocotyl elongation in *Arabidopsis* and other plants (Wang et al., 2020). Our results show that at least two *SAURs* are direct targets of GmPIF1 and GmPIF3 (Figures 3D,E). PIFs are involved in light and auxin regulating hypocotyl elongation pathways in a variety of plants (Sun et al., 2020). It is not surprising that we found that the expression of *GmSAUR1, GmSAUR23*, and *EXPANSIN*s were all regulated by auxin and light (Figure 4; Supplementary Figure 9). Based on these results, we constructed the auxin/light- GmPIF1/GmPIF3-GmSAUR1/23- EXPANSINs regulatory submodule, in which auxin/light regulates *GmSAUR1* and *GmSAUR23* through GmPIF1 and GmPIF3, and in turn regulates hypocotyl elongation through EXPANSINs (Figure 3C; Supplementary Figure 15). Interestingly, in *Arabidopsis thaliana*, light promoted the expression of *SAURs* (*SAUR14, SAUR16*, and *SAUR50*), while auxin inhibited (Dong et al., 2019). But in soybean we observed the opposite regulation pattern, i.e., auxin promoted *SAURs*' expression while light inhibited (Figure 4), which may be due to the functional differentiation of SUAR genes or the difference between species.

Plant hypocotyl elongation is mainly regulated by transcription factors. There are 83 transcription factors in cyan module, most of which are enriched in auxin and light signal response pathway (Supplementary Figure 10; Figure 5A). We note that four *GmPRE6* genes and several key regulators closely related to them form a regulatory submodule (Figure 5B). In *Arabidopsis*, PRE6 is involved in the hypocotyl elongation regulated auxin signaling, but the mechanism remains largely elusive (Hong et al., 2013; Zheng et al., 2017). Interestingly, there is a homologous submodule in the *Arabidopsis* regulating hypocotyl elongation (Figure 5C), implying the conservation of relative mechanisms. The expression level of *GmPRE6*s in soybean hypocotyl was induced by exogenous auxin and repressed by light, indicating they may act downstream to regulate hypocotyl elongation (Figure 5D; Supplementary Figures 14A–D). Indeed, ectopic expression of *GmPRE6s* in *Arabidopsis* led to abnormal hypocotyl elongation (Figure 6A). Interestingly, by analyzing the ChIP-seq data of *Arabidopsis thaliana* (Pfeiffer et al., 2014), we found that PIF3 can bind to the *PRE6* promoter (Supplementary Table 5). The analysis of soybean *GmPRE6* promoters showed that there are auxin/light response elements and G-box elements (Supplementary Figure 14E), indicating that *GmPRE6s* are potential targets of GmPIFs. Therefore, GmPRE6s are promoters of hypocotyl elongation, and light stimulus inhibit their expression to repress hypocotyl elongation, while auxin plays a opposite role (Supplementary Figure 15). GmPRE6s were expressed in the nucleus, but they were reported to be atypical bHLH TFs that lack the basic DNA binding domain, so they may regulate downstream processes by combining with other TFs. In *Arabidopsis*, PRE6 forms non-functional heterodimers with HFR1 to activate PIFs. In the GmPRE6s submodule, there is a homolog of HFR1, GmPIL15. So GmPRE6s may interact with GmPIL15 to induce the elongation of hypocotyl (Supplementary Figure 15). Future studies could be carried out to clarify the mechanisms.

In conclusion, through WGCNA analysis, we constructed the global regulatory network of soybean hypocotyl elongation for the first time, and identified a key regulatory module. By combining with previous research results, as well as transcription factor analysis, Y1H, mutant and transgenic plant phenotype analysis, we identified two regulatory modules, namely *GmPRE6s-*EXPANSINs submodule and GmPIF1/GmPIF3-GmSAUR1/23- EXPANSINs submodule, respectively (Supplementary Figure 15). Our results not only reveal the key regulatory network of hypocotyl elongation, but also narrow down the gene range of hypocotyl elongation regulation, providing valuable gene resources for improving soybean hypocotyl shape and seed vigor through soybean breeding.

## Data Availability Statement

The original contributions presented in the study are publicly available. This data can be found here: National Center for Biotechnology Information (NCBI) BioProject database under accession number SRR17239198. https://www.ncbi.nlm.nih.gov/sra/?term=%20SRR17239198.

## Author Contributions

MC conceived the study and supervised the whole research. ZS performed experiments and analyzed the data. MC and ZS wrote the manuscript. All authors approved the submitted version.

## Funding

This work was supported by the National Natural Science Foundation of China (31970344) to MC.

## Conflict of Interest

The authors declare that the research was conducted in the absence of any commercial or financial relationships that could be construed as a potential conflict of interest.

## Publisher's Note

All claims expressed in this article are solely those of the authors and do not necessarily represent those of their affiliated organizations, or those of the publisher, the editors and the reviewers. Any product that may be evaluated in this article, or claim that may be made by its manufacturer, is not guaranteed or endorsed by the publisher.
