# Supplementary material for: Deciphering Novel Transcriptional Regulators of Soybean Hypocotyl Elongation Based on Gene Co-expression Network Analysis
Source: Front Plant Sci. 2022 Feb 22;13:837130. doi: 10.3389/fpls.2022.837130 (PMC8902393; doi:10.3389/fpls.2022.837130)
Supplement: Supplementary file 2 [file Data_Sheet_1.PDF]

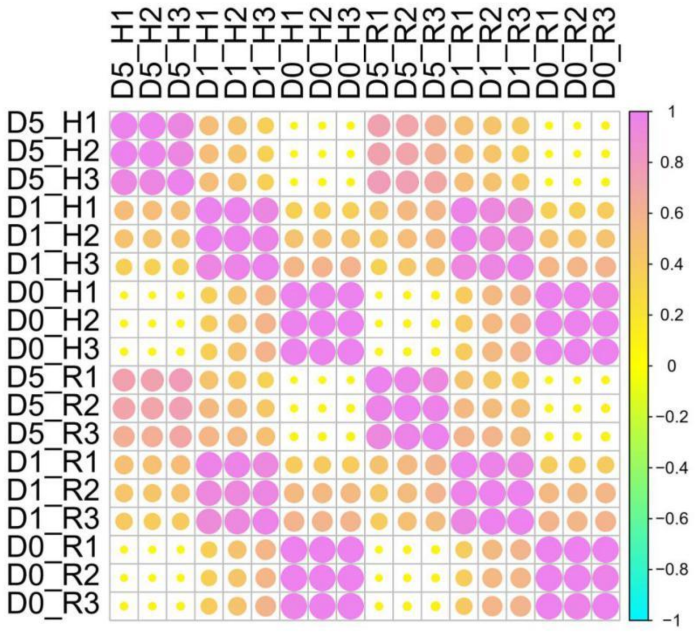

Supplementary Figure 1 Correlation between samples used for WGCNA. The dot size indicates the correlation coefficient value between the two samples, while the color indicates the correlation direction: Pink- positive correlated, yellow- uncorrelated, cyan- negative correlated.

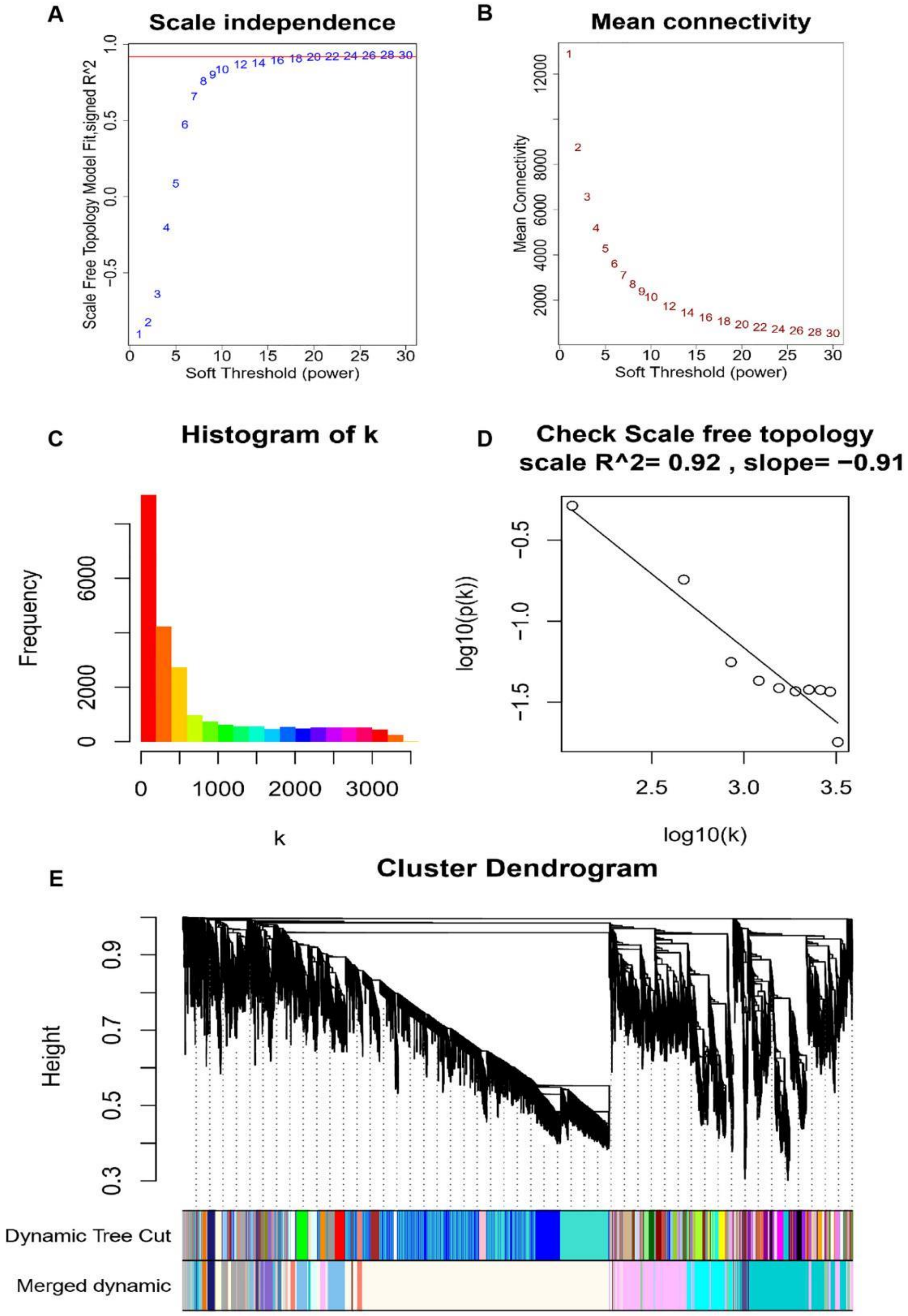

Supplementary Figure 2 WGCNA module construction. (A, B) The soft-thresholding power (X-axis) was set to 18 to ensure a scale-free network. (C, D) Histogram showing distribution of the connectivity of all genes. (E) Clustering dendrogram obtained by hierarchical clustering of adjacency-based dissimilarity. The upper color bar indicates "Dynamic Tree Cut" results and the lower indicates merged results using the parameter PCC > 0.85

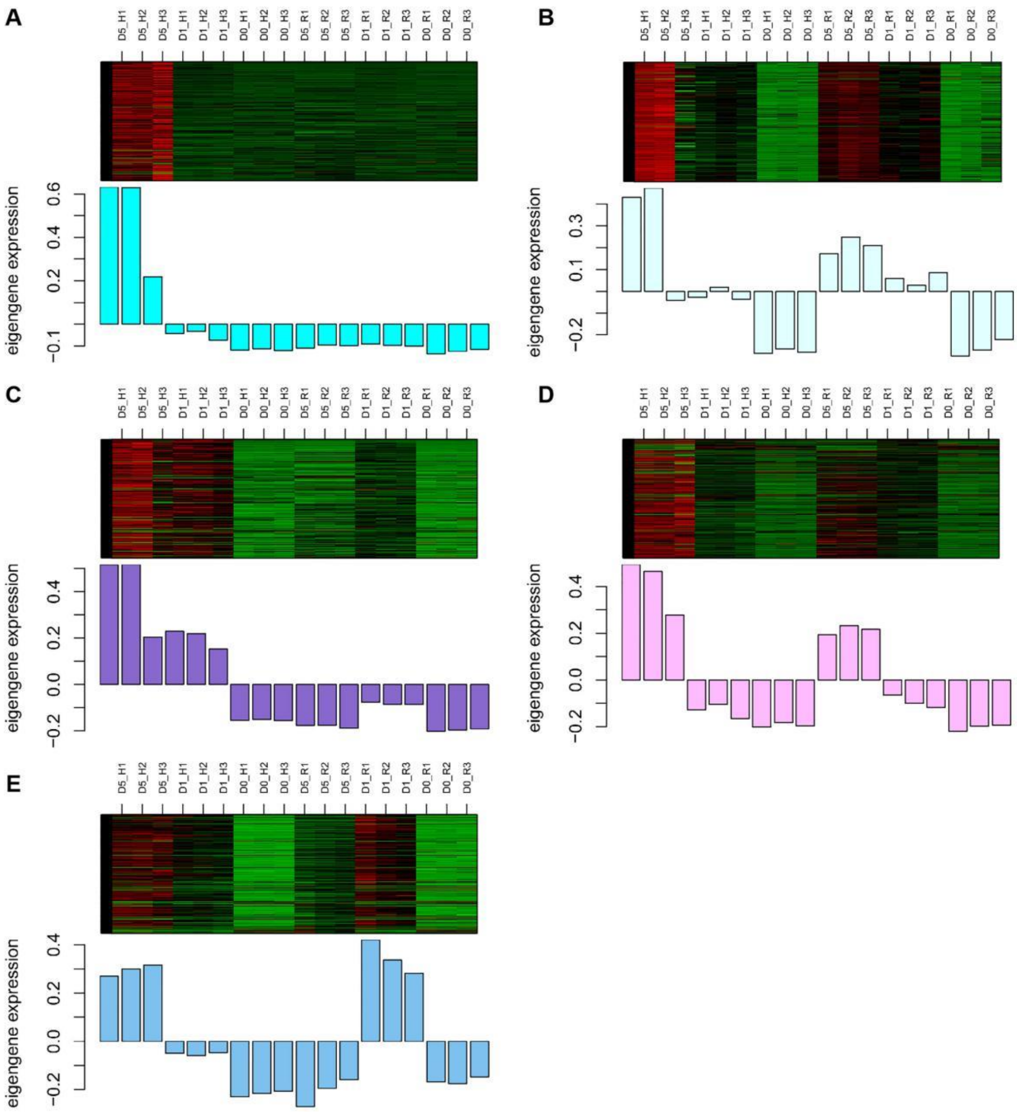

Supplementary Figure 3 Expression pattern of genes from the five-hypocotyl elongation related modules. (A) “cyan” module. (B) “lightcyan” module. (C) “mediumpurple3” module. (D) “plum1” module. (E) “skyblue2” module. In the heatmap, green indicates downregulation, and red indicates upregulation. The histograms indicate the corresponding module eigengene expression value (y-axis) of each sample. H: hypocotyl, R: root tip.

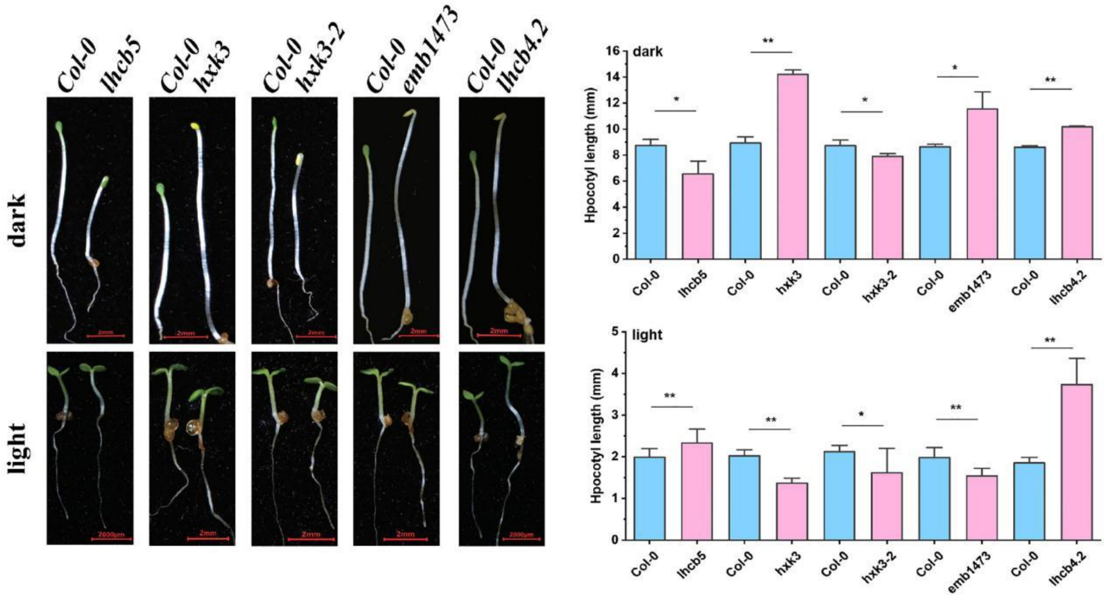

Supplementary Figure 4 Hypocotyl elongation phenotype of Arabidopsis mutants. Data are presented as mean ± SD (n≥10). A statistically significant difference of $p < 0.05$ or $p < 0.01$ relative to mock (two-tailed Student's t test) is denoted by * and **, respectively.

```
Glyma.13G130100   RAAEVHNLSERRRRDRINEKMKAIQELIPRCNKSDKASMLDEAIEYIKSIQLQVQ....M    56
atPIF1            RAAEVHNLSERKRRDRINERMKAIQELIPRCNKSDKASMLDEAIEYMKSIQLQIQ....M    56
Glyma.19G222000   RNPEVHNLSEKKRREKINKKMRTLKDLIPNCNKVDKASMLDDAIDYIKTIKLQLQANFQI    60
atPIF3            RSAEVHNLSERRRRDRINEKMRALQELIPNCNKVDKASMLDEAIEYIKSIQLQVQ....I    56
Consensus         r  evhnlse  rr  in  m  l  lip cnk dkasmld ai  y k l lq q

Glyma.13G130100   MSMGYCM    63
atPIF1            MSMGC..    61
Glyma.19G222000   MSMGSGL    67
atPIF3            MSMASGY    63
Consensus         msm
```

Supplementary Figure 5 Alignment of PIF1 and PIF3 bHLH domain sequence in soybean and *Arabidopsis thaliana*.

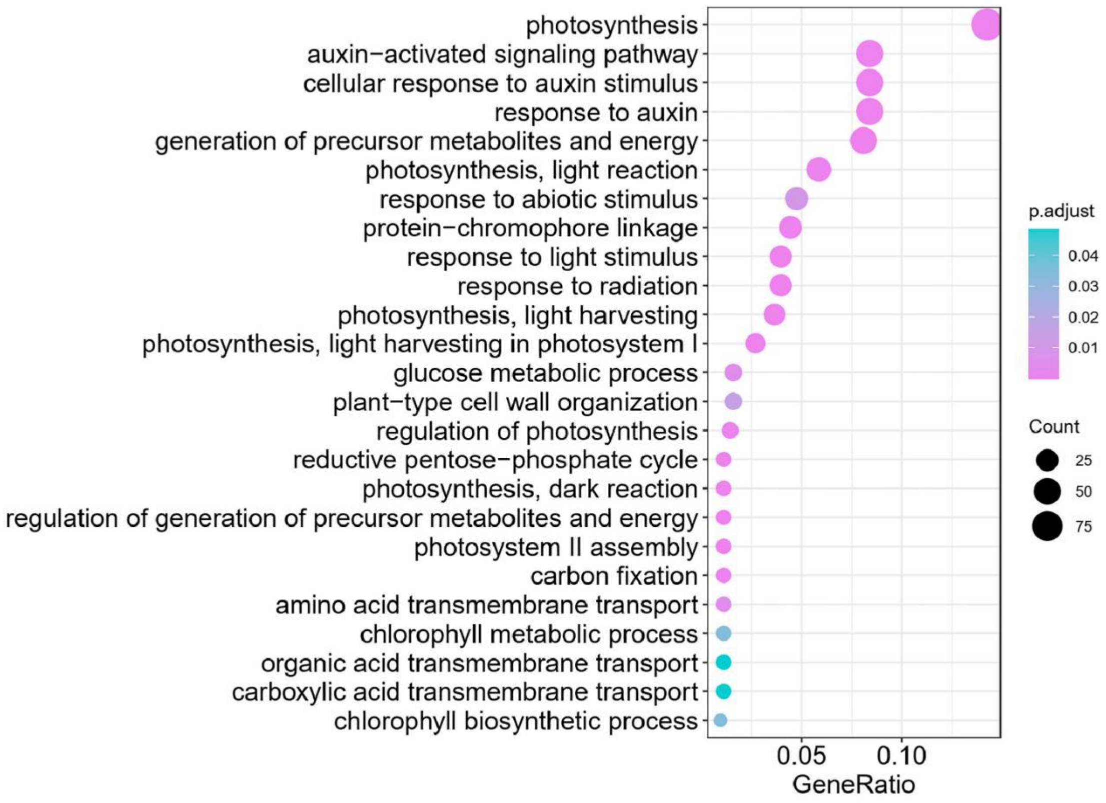

Supplementary Figure 6 GO analysis of genes in cyan module. The dot size indicates the number of genes, while the dot color indicates the p value: pink- lower p value, cyan- higher p value.

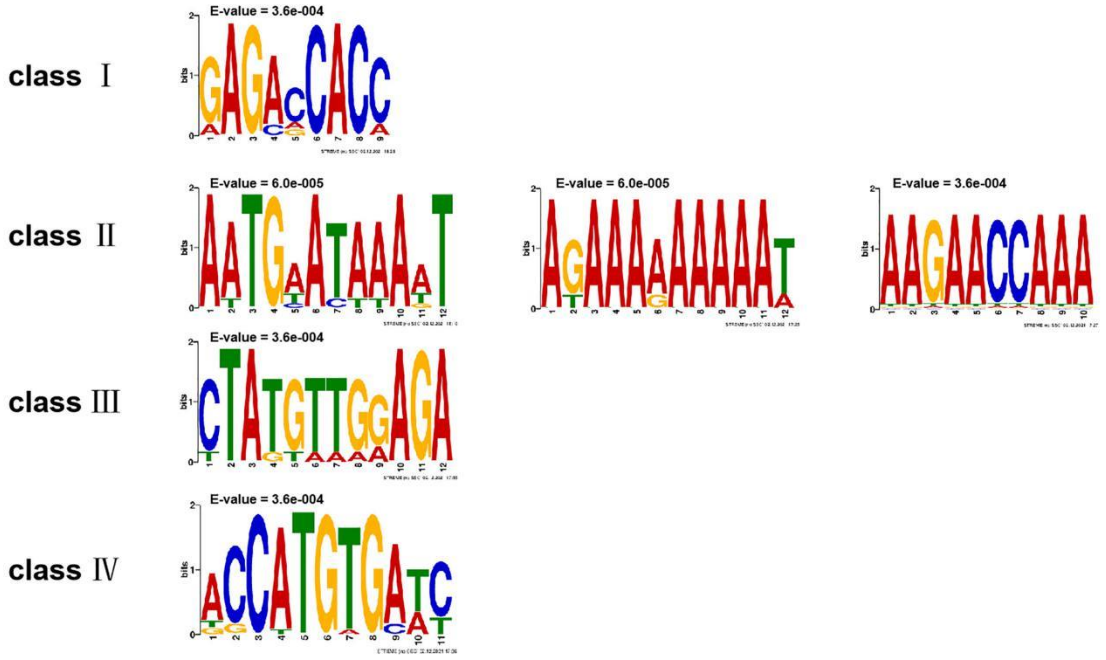

Supplementary Figure 7 cis-element prediction in the promoters of the 27 soybean genes and their homologs in *Arabidopsis*. Class I represents auxin response element, class II represents light response element, class III represents RAV1-AP2-VP1 binding element, and class IV represents dehydration response element.

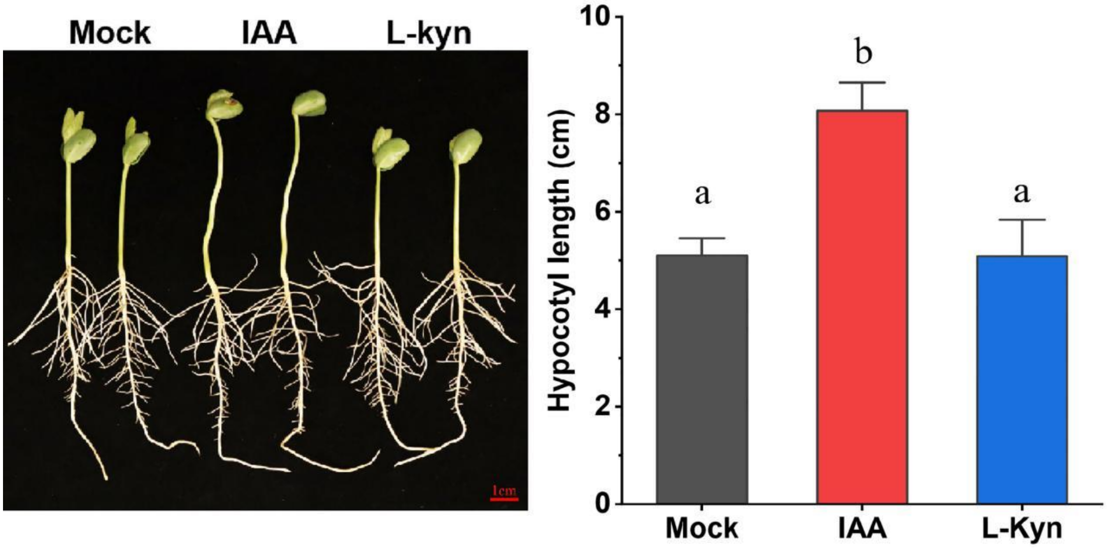

Supplementary Figure 8 Effect of exogenous IAA and Kyn on soybean hypocotyl elongation. Soybean seedling were under light condition. Data are presented as mean ± SD (n=6-8). Different letters (a, b) indicate significant difference (two-tailed Student's t test).

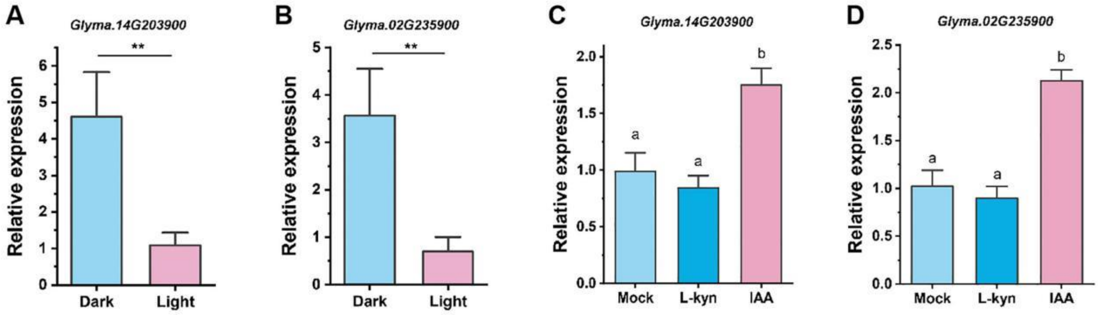

Supplementary Figure 9 Expression of two *EXPANSINs* in response to light and exogenous auxin. (A, B) Expression of *EXPANSINs* in soybean hypocotyl under light and dark condition. (C, D) Expression of *EXPANSINs* in the hypocotyl after 2 h treatment of exogenous IAA and Kyn. Data are presented as mean ± SD (n = 3). A statistically significant difference of $p < 0.05$ or $p < 0.01$ relative to mock (two-tailed Student's t test) is denoted by * and **, respectively.

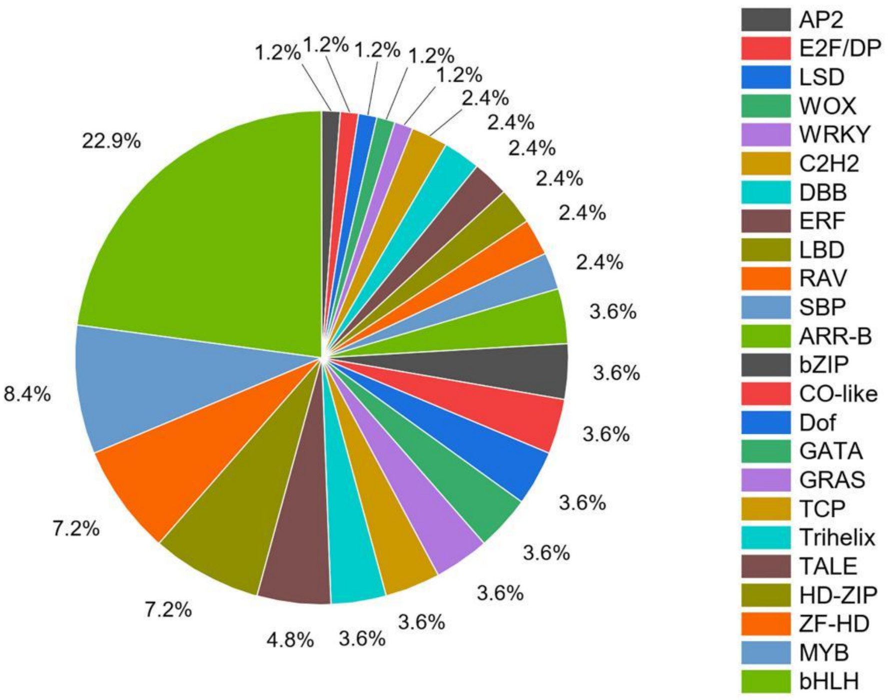

Supplementary Figure 10 Prediction of transcription factors in the cyan module.

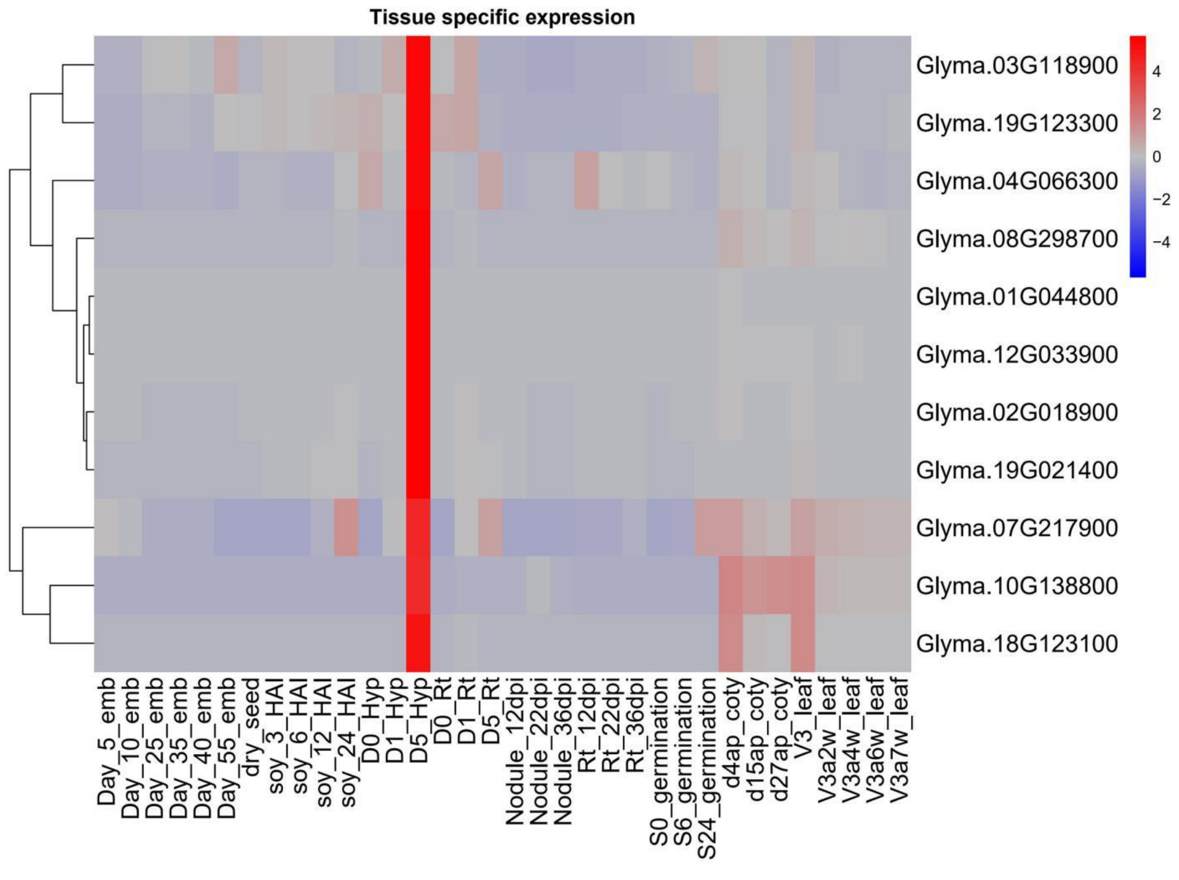

Supplementary Figure 11 Expression pattern of genes from the GmPRE6s submodule in different soybean tissues. HAI: hours after imbibition, emb: embryo, Hyp: hypocotyl, Rt: root, dpi: days post inoculation, coty: cotyledon.

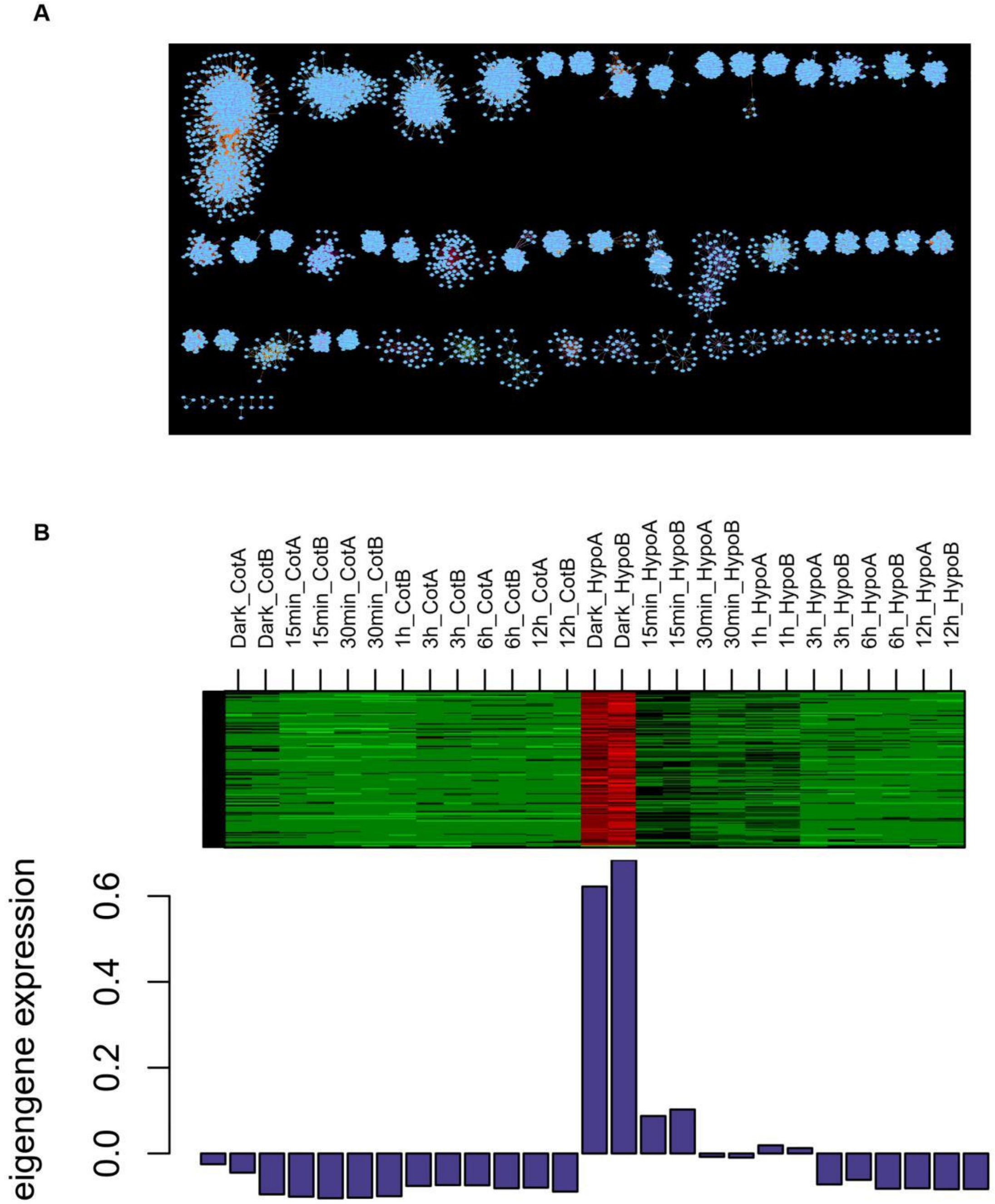

Supplementary Figure 12 A global gene co-expression network related to hypocotyl length in *Arabidopsis*. (A) Global gene co-expression network including 30 modules visualized with Cytoscape. (B) Expression pattern of genes from the five-hypocotyl elongation related modules. In the heatmap, green indicates downregulation, and red indicates upregulation. The histograms indicate the corresponding module eigengene expression value (y-axis) of each sample. Hyp: hypocotyl, Cot: cotyledon

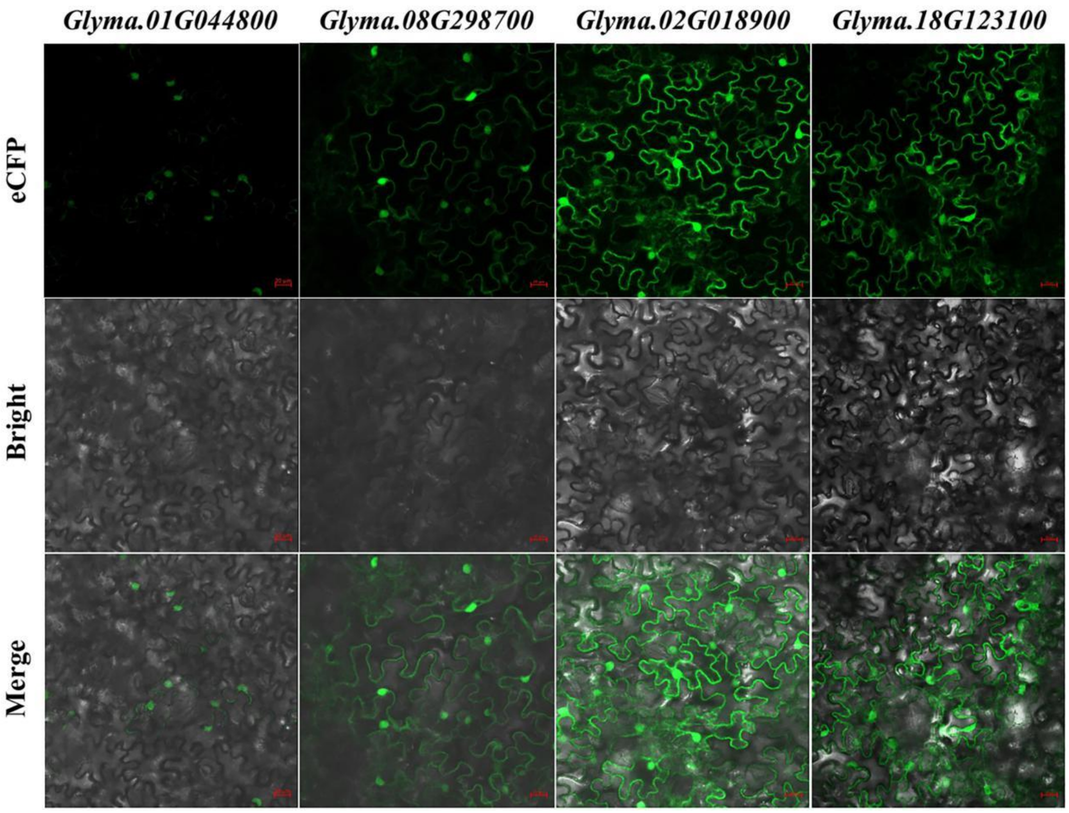

Supplementary Figure 13 Subcellular localization of four GmPRE6s fused eCFP in nuclei and plasma membrane. Scale bars=20 μm.

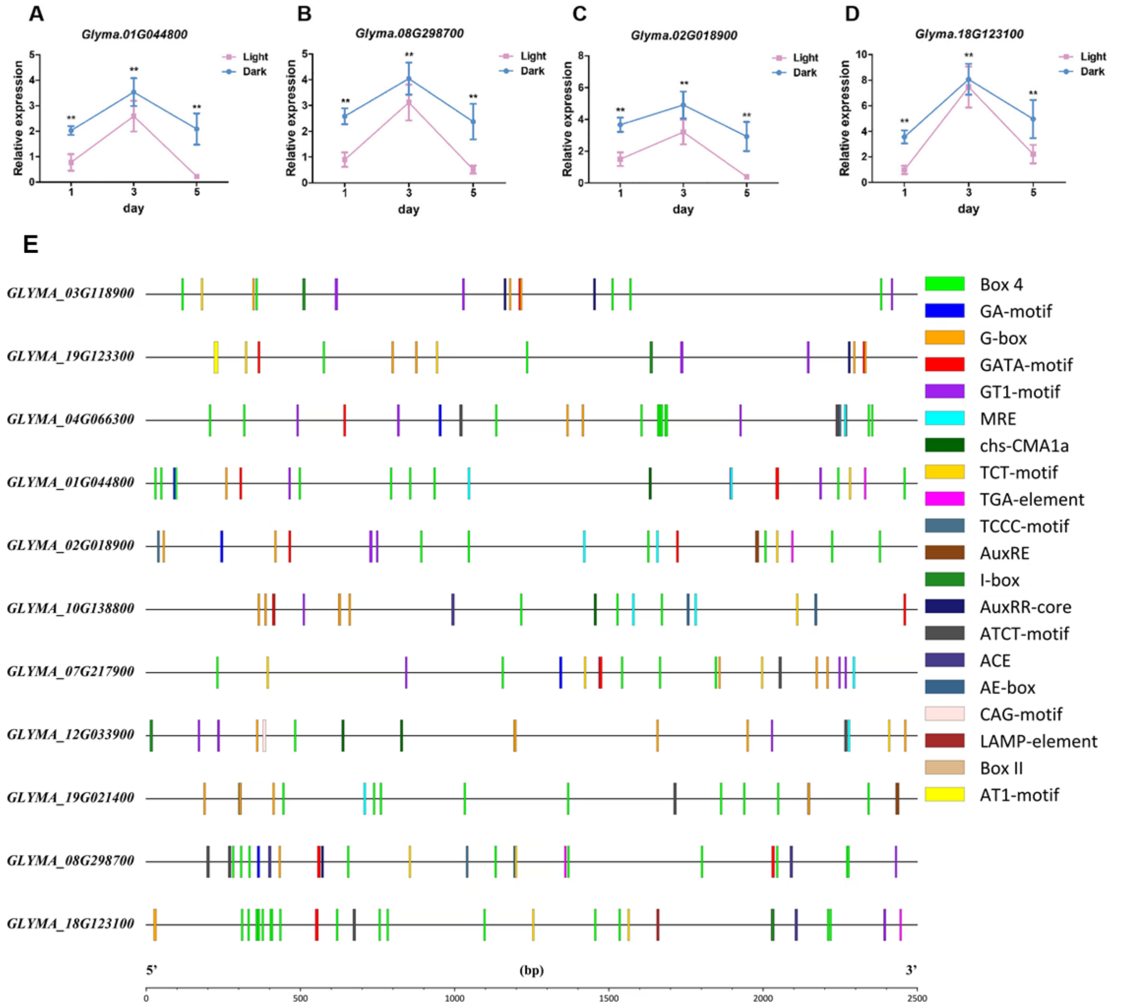

Supplementary Figure 14 Expression of four GmPRE6 genes in response to light signal. (A-D) Expression of four GmPRE6 genes in soybean hypocotyl under light and dark condition. A statistically significant difference of $p < 0.05$ or $p < 0.01$ relative to mock (two-tailed Student's t test) is denoted by * and **, respectively. (E) Cis elements in the promoter of *GmPRE6s* and related genes, including light response elements: G-box, Box4, GATA-motif, GA-motif, chs-CMA1a, TCT-motif, GT1-motif, MRE, TCCC-motif, I-box, ATCT-motif, TCCC-motif, ACE, AE-box, CAG-motif, LAMP-element and AT1-motif, and auxin response elements: TGA-element, AuxRR-core and AuxRE.

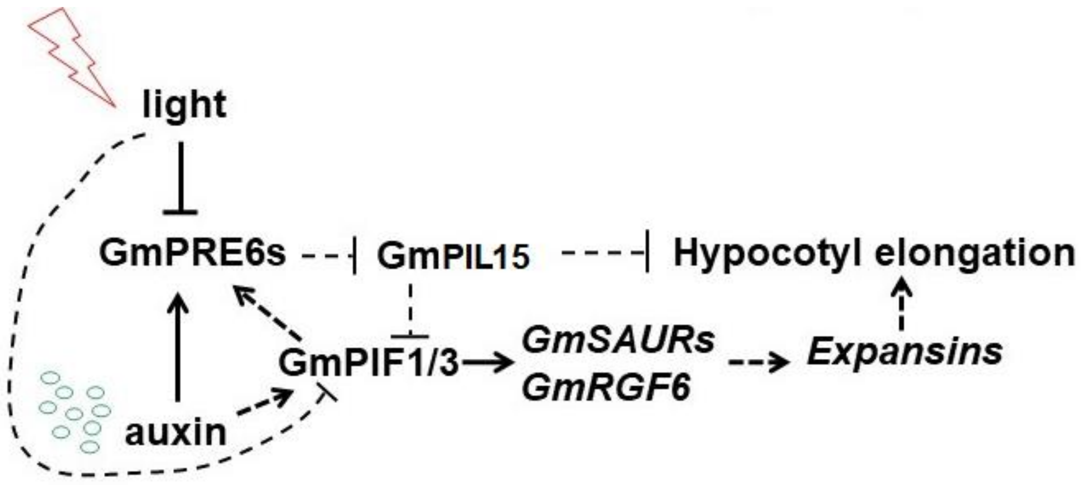

Supplementary Figure 15 A working model for the regulation of soybean hypocotyl elongation. Auxin induces the expression of *GmREF6* and further promotes hypocotyl elongation through downstream factors such as GmPIL15, while light inhibited *GmREF6* expression to repress hypocotyl elongation. In addition, light and auxin may regulate hypocotyl elongation through GmPIF1/3 and its downstream elements. There is interaction between the two pathways.
